# Best Water Vapor Information Layer of Himawari-8-Based Water Vapor Bands over East Asia

**DOI:** 10.3390/s20082394

**Published:** 2020-04-23

**Authors:** You Wu, Feng Zhang, Kun Wu, Min Min, Wenwen Li, Renqiang Liu

**Affiliations:** 1 Key Laboratory of Meteorological Disaster, Ministry of Education (KLME)/Collaborative Innovation Center on Forecast and Evaluation of Meteorological Disaster (CIC-FEMD), Nanjing University of Information Science and Technology, Nanjing 210044, China; 20181201080@nuist.edu.cn (Y.W.); wukun@nuist.edu.cn (K.W.); liwenwen0213@nuist.edu.cn (W.L.); rq_liu@nuist.edu.cn (R.L.); 2 Department of Atmospheric and Oceanic Sciences & Institute of Atmospheric Sciences, Fudan University, Shanghai 200438, China; 3 School of Atmospheric Sciences and Guangdong Province Key laboratory for Climate Change and Natural Disaster Studies, Sun Yat-Sen University and Southern Laboratory of Ocean Science and Engineering (Guangdong, Zhuhai), Zhuhai 519082, China; minm5@mail.sysu.edu.cn

**Keywords:** best water vapor information layer, water vapor remote sensing, ERA-interim dataset assessment

## Abstract

The best water vapor information layer (BWIL), based on Himawari-8 water vapor bands over a typical region of East Asia, is investigated with the U.S. standard atmospheric profile and European Centre for Medium-Range Weather Forecasts Re-Analysis-interim (ERA-interim) dataset. The sensitivity tests reveal that the height of the BWIL is connected heavily to the amount of water vapor in the atmosphere, and to the satellite zenith angle. According to the temporal and spatial distribution analysis of BWIL, there are two basic features of BWIL. First, it lifts from January to July gradually and descends from July to October in the whole region. Second, it is higher over sea than land. These characteristics may stem from the transport of water vapor by monsoon and the concentration of water vapor in different areas. With multiple water vapor absorption IR bands, Himawari-8 can present water vapor information at multiple pressure layers. The water vapor content of ERA-interim in July 2016 is assessed as an example. By comparing the brightness temperatures from satellite observation and simulation under clear sky conditions, the ERA-interim reanalysis dataset may underestimate the amount of water vapor at pressure layers higher than 280 hPa and overestimate the water vapor quantity at pressure layers from 394 to 328 hPa, yet perform well at 320~260 hPa during this month.

## 1. Introduction

Although the proportion of water vapor (WV) in the atmosphere is very small (only 1–4% by volume), it is not only an important greenhouse gas but also the only component in the atmosphere that can change phases. Thus, water vapor is significant in severe weather forecasts and in maintaining a global energy balance [1,2,3,4]. The infrared 6–7 μm band is the most important water vapor absorption spectrum, and it is often selected to receive water vapor radiation information for geostationary satellites. For example, the European Meteosat-1 launched in 1977 is the first geostationary satellite throughout the world that can sense water vapor, and the Meteosat series carry a 5.7–7.1 μm water vapor (WV) absorption band [5]. The first Chinese geostationary satellite FengYun (FY)-2B (launched in 2000) carries a 6.3–7.6 μm WV band [6]. With the development of satellite detection capability, multiple-WV-bands-carrying satellites emerged, such as the Chinese FY-4 and Japanese Himawari-8 [7,8]. Himawari-8, launched on 17 October 2014, is in geostationary orbit at a height of 35,800 km. The operation of the Himawari-8 started in July 2015, and the utilization of this new GEO satellite shows promise in weather prediction, disaster prevention and scientific studies of atmospheric, ocean and land processes. The Himawari-8 carries a multispectral imager, the Advanced Himawari Imager (AHI), which has 16 spectral bands in visible (VIS), near-infrared (NIR) and thermal infrared (TIR) spectra. The spatial resolution has been significantly improved (0.5–2 km at the nadir). In addition, the advantages of the Himawari-8 are not only an increase in spectral bands and spatial resolution but also a capability for frequent observations. It scans the full disk of the globe every 10 min and selected regions every 2.5 min [9]. The frequent observations add a new dimension in satellite observation and open a new science of the atmosphere and weather system [10]. Himawari-8 satellite data has been widely used in the meteorology and radiation field due to its high spatial-temporal resolution and good accuracy, including a novel machine-learning (ML) algorithm to retrieve the cloud top height using Himawari-8 [11], estimating summertime precipitation from Himawari-8 and the Global Forecast System (GFS) based on ML [12], estimating ground-level PM2.5 concentrations in central China [13], tracking local severe storms from Himawari-8 by the Random Forest (RF) algorithm [14], analyzing the spatial and temporal distribution and variation of the Aerosol Optical Depth (AOD) in coastal southeast China [15], and so forth. AHI also has a strong capability for water vapor detection, with three water vapor bands centered at 6.21 μm, 6.93 μm and 7.34 μm, respectively.

The Jacobian function of water vapor $\left(\frac{dT_{b}}{dlnq}\right)$ is often used to discuss the change of the brightness temperature caused by a slight change of water vapor in a certain pressure layer. Meanwhile, it has a great reference value for comprehending the abilities and limitations of a given band [16]. What is more, the Jacobian function not only helps to retrieve the water vapor profile [17,18] but is also a significant parameter in satellite radiance data assimilation [19]. Zeng [20] defined the best water vapor layer (BWIL) for representing the corresponding pressure layer of the minimum value of the water vapor Jacobian function in a satellite water vapor absorption band. Naturally, multiple WV bands will match various BWILs, but often only one water vapor band can be given in most cases because of limited technology in the past. Besides, the lowest height of BWIL usually reaches the middle-upper troposphere, which indicates that the information of water vapor can only be obtained in the middle-upper troposphere, especially in the upper troposphere [21,22,23,24,25,26]. Concerning specific studies on BWIL, Poc et al. [27] did some studies over the Mediterranean and Western European regions, suggesting that the peak height of the contributions to the radiation varies from about 550 hPa to approximately 450 hPa. Additionally, Di et al. [28] analyzed the BWIL of the 6.7 μm water vapor absorption band for the FengYun-2E over the Tibetan Plateau and the East China Plain and pointed out that surface characteristics can influence BWIL to some extent. However, most previous studies are aimed at one WV channel and rarely involve East Asia, which causes a limited understanding of more information about water vapor at different pressure levels in the atmosphere. In particular, the radiance information of water vapor in the lower troposphere is rarely explored with other WV bands. For this limitation, the investigations on multiple WV bands of AHI will help to understand the detection capability of water vapor at different heights, which may become an indication for water vapor data assimilation in numerical weather prediction (NWP). In this paper, we will study the BWIL of Himawari-8 based water vapor bands over East Asia, in which recent frequent occurrences of heavy rainfall events prompted public attention and demands for the monitoring of severe weather and a better understanding of the mechanism.

The model and data used are illustrated in Section 2. Section 3 mainly describes the impact factors (water vapor amount, satellite zenith angle and surface IR emissivity) on BWIL and analyzes the seasonal and the land-sea variations of BWIL over a typical region of East China. Section 3 also shows an assessment work of ERA-interim water vapor in July 2016. The summary and some prospects for future work are written in the last section.

## 2. Model and Data

**Figure 1:**
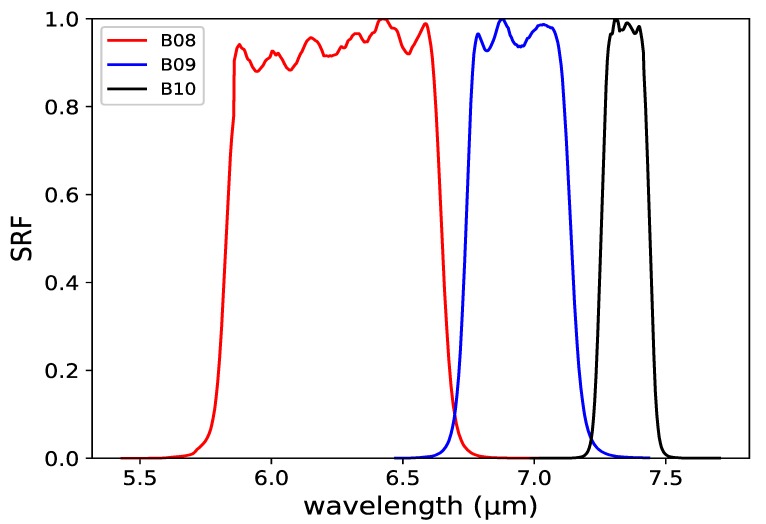
SRFs of the water vapor absorption IR bands for AHI.

In WV bands, the satellite can measure the radiation emitted by the earth-atmosphere-system including the radiative effect of WV absorption and emission, which becomes the basic principle for sensing water vapor by satellite. The Satellite Response Functions (SRFs) and corresponding wavelengths for the three WV bands are shown in Figure 1.

**Figure 2:**
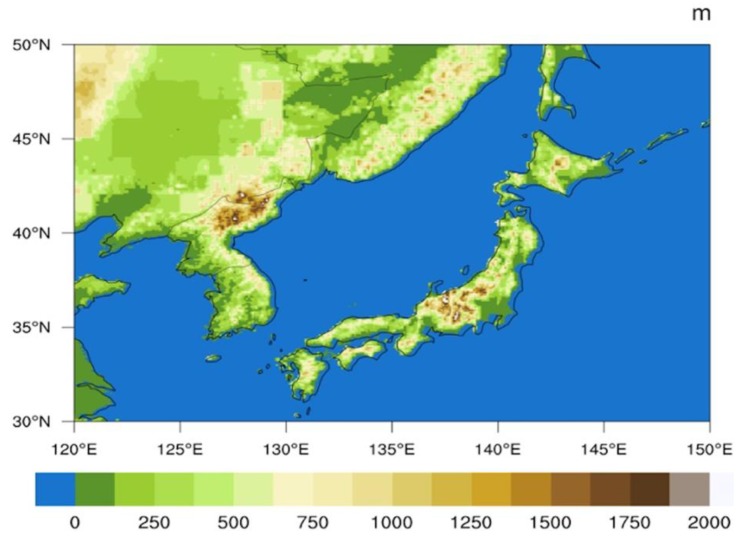
Elevation distribution over the typical region of East Asia. Units are m.

To accurately capture the real features of water vapor, bright temperatures are simulated under a clear sky condition by an alternate mapping correlated k-distribution (AMCKD) method [29] to achieve gas absorption parameterization. It is based on the rearrangement of the gaseous absorption coefficient in each portion of cumulative probability space (CPS) to capture the main characteristics of each gas, and it turns out to be efficient and accurate for AHI on board Himawari-8 simulations. The input variables include the temperature, water vapor concentration, ozone atmospheric vertical profiles and surface skin temperature, which are from the ERA-interim data set [30]. Compared with the ERA-40 published before, the ERA-interim dataset has been improved in terms of resolution, physical parameters of the model, four-dimensional variational method, error correction and so on. The dataset used in this study is accessible every 6 h (00, 06, 12 and 18 UTC) with a spatial resolution of 0.5°×0.5° and a vertical resolution of 37 pressure layers from 1000 hPa to 10 hPa. The surface IR emissivity is the land 8-day mean level 3 product (MYD11C2) from the Moderate Resolution Imaging Spectroradiometer (MODIS) [31,32,33]. In ocean areas, the surface IR emissivity is set to 0.99. The satellite-observed brightness temperatures applied for comparison in this study are the Himawari level 1 gridded data with a temporal resolution of 10-min and a spatial resolution of 0.05° × 0.05°. Our study focuses on Himawari-8 WV bands centered at 6.21 μm, 6.93 μm and 7.34 μm over a typical region of East Asia (30–50° N and 120–150° E), including the Northeast Plain of China, Korean Peninsula, Japan, and some sea areas. The map in Figure 2 shows the location of the selected typical region as well as the elevation. The U.S. standard atmospheric profile [34] with 400 levels is used in the sensitivity tests, and the analysis of Jacobian functions over the selected region is calculated with each ERA-interim reanalysis profile in January, April, July and October 2016 by AMCKD. We also analyze the seasonal variations and land-sea differences in the height of BWIL and the magnitude of Jacobian functions based on the ERA-interim reanalysis profiles. Furthermore, we use a reliable cloud product with a spatial resolution of 2 km ×2 km and a temporal resolution of 6 h [35] to eliminate cloudy grids for assessment in Section 3. This product, with hit rate (HR) values of about 93% for AHI, is highly qualified to retrieve real-time cloud masks during both daytime and nighttime.

## 3. Results and Discussion

### 3.1. Impact Factors on BWIL

It has been proven that the water vapor Jacobian function depends on the vertical water vapor profile and viewing angle, yet the sensitivity to temperature changes in the atmosphere is relatively small based on METEOSAT and the Nimbus/THIR single water vapor channel [36]. To understand the basic features of BWIL and the Jacobian function of the three WV bands on AHI, three sensitivity tests are designed to explore the impact factors of BWIL, in which the water vapor mass, the viewing angle and the surface IR emissivity are taken into account. The formula of the Jacobian function $w_{q}\left(p\right)$ can be written as the following equation according to Li et al. [37]:

(1) $$w_{q}\left(p\right)=\frac{dT_{b}}{dlnq}\approx \frac{T_{b2}-T_{b1}}{lnq_{2}-lnq_{1}}$$

where $q_{1}$ represents the water vapor concentration at a barometric layer in the original atmospheric profile. Notably, the unit of $q_{1}$ is Kg/Kg , and the unit of $w_{q}$ is K/log(Kg · Kg)^−1^ $q_{2}$ is a slight change in $q_{1}$ Correspondingly, $T_{b1}$ means the simulated brightness temperature using the initial atmospheric profile, and $T_{b2}$ is the simulated brightness temperature after a slight change of the water vapor content at a certain level. In this study, we set the increment of the water vapor concentration at 1.1 times compared with the initial atmospheric profile. This calculation method, which is original but computationally efficient, is most in line with the physical meaning of the Jacobian function. 

**Figure 3:**
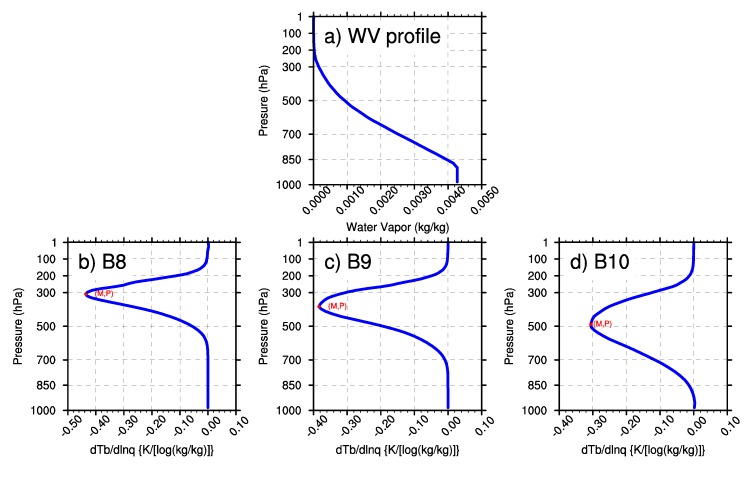
(**a**) The water vapor profile of the U.S. standard atmosphere. (**b**)–(**d**) The calculated Jacobian function of Band 8, Band 9 and Band 10, respectively.

In this research, the minimal values of $w_{q}$ and the BWILs of the three WV bands are analyzed based on the U.S. standard atmospheric profile. In Figure 3, M represents the minimum value of the Jacobian function; P represents the height of BWIL (measured by pressure layer) when the value of the Jacobian function reaches the minimum value. Figure 3a shows the water vapor profile that was input to AMCKD, and Figure 3b-d illustrate the Jacobian function of Band 8, Band 9 and Band 10 to apply the geostationary satellite in a better way, respectively. In Figure 3b–d, the height of BWIL decreases from Band 8 to Band 10: for Band 8, the height of BWIL is about 300 hPa, and it increases to about 400 hPa for Band 9, while it is around 500 hPa for Band 10. The height of BWIL closely links to the strength of the absorption effect. Specifically, the stronger the absorption effect is, the larger the optical depth is, and the more severely the WV radiation information from lower layers decays when it travels upward. Because of the strongest WV absorption effect of Band 8 among the three WV bands, the height of BWIL for Band 8 rises the highest. The value of M increases from Band 8 (about –0.44) to Band 10 (about –0.31). Generally, the value of M is negative because water vapor usually blocks the view of the satellite sensor [28], but for convenience, we will discuss the numerical value of the minimum value of M (|M|) in the following sections. It should be noted that a larger |M| means a bigger moisture sensitivity and better remote sensing capability for the corresponding BWIL height, so Band 8 can sense WV best among the three bands. 

**Figure 4:**
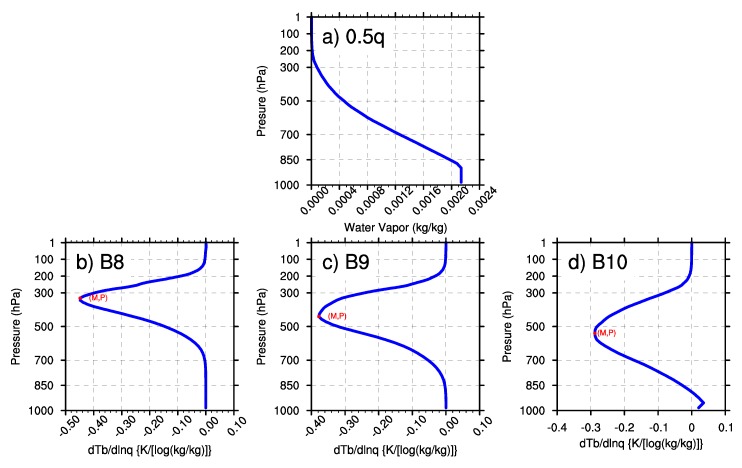
(**a**) Half of the water vapor content in Figure 3a. (**b**)–(**d**) Corresponding Jacobian functions (M) and the height of BWIL (P) of Band 8, Band 9 and Band 10, respectively.

**Figure 5:**
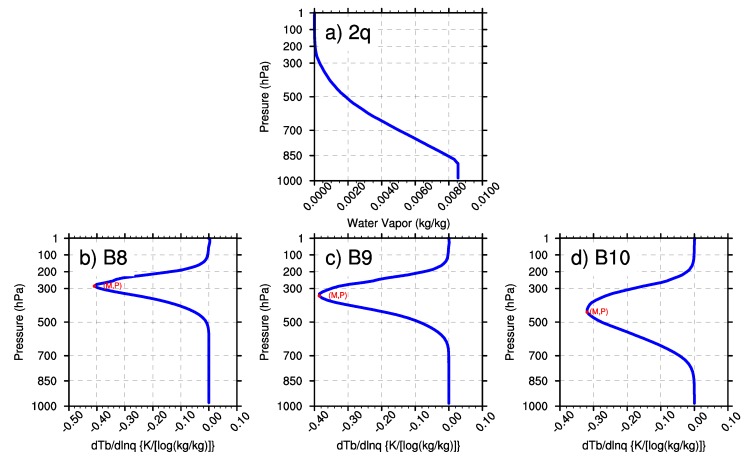
(**a**) Double water vapor content in Figure 3a. (**b**)–(**d**) Corresponding Jacobian Functions (M) and the height of BWIL (P) of Band 8, Band 9 and Band 10, respectively.

The first sensitivity test changes the water vapor content while keeping the surface IR emissivity at 0.99 and the surface skin temperature at 288 K. Figure 4a and Figure 5a illustrate the WV profiles of multiplying the U.S. standard atmosphere WV profile by 0.5 and 2, respectively. Then, the corresponding M and P of the three WV bands are revealed in Figure 4b–d and Figure 5b–d for the three WV bands. It can be obtained that the height of BWIL for all three WV bands increases gradually with the increase of the water vapor content. For the reason discussed above, more water vapor content will result in the larger optical depth and higher height of BWIL. Concerning the value of |M|, the three bands perform quite differently: the value of |M| decreases when the water vapor mass increases for Band 8, while it is almost unchanged for Band 9. For Band 10, |M| varies synchronously with the change of the water vapor content. Similarly, the absorption effects of the different bands also play a key role. It is known from Figure 3b that the height of BWIL of Band 8 is the highest of the three bands: about 300 hPa. However, due to a little water vapor amount at this pressure layer and the strongest absorption effect of Band 8, when the water vapor mass increases to a certain degree, the absorption of water vapor for infrared radiation will reach saturation point. This will lead to a lower sensitivity to the water vapor content of Band 8 and a smaller |M|. Instead, Band 10 has the weakest absorption effect, which results in a higher sensitivity and larger |M| as the water vapor content increases.

**Figure 6:**
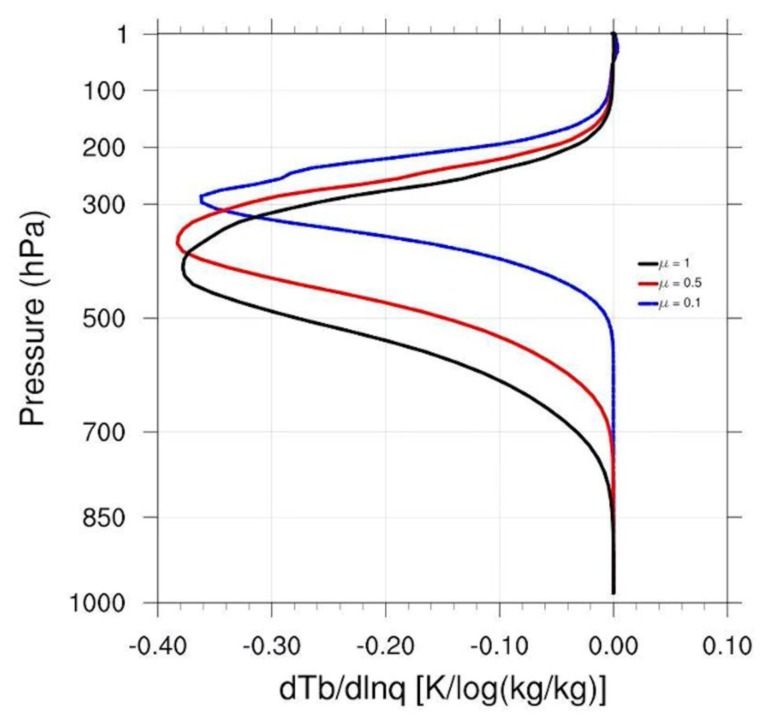
Calculated Jacobian functions of Band 9 under different SAZs (black line for 0°, red line for 60°, and blue line for about 84.26°).

Apart from the water vapor content, the satellite zenith angle is also a significant impact factor for BWIL. To explore this, we use the U.S. standard atmospheric profile and take Band 9 as an example, setting the cosine of the satellite zenith angle μ to 0.1, 0.5 and 1, respectively. As is displayed in Figure 6, the smaller μ is, the higher the BWIL will be. This is because the smaller the μ is, the longer the path of the radiation information to the satellite will be and, accordingly, the larger the optical depth will be.

To investigate the effect of the surface IR emissivity (ε) the BWIL, we set it from 0.6 to 1.0 with an interval of 0.1. Meanwhile, the surface skin temperature is kept as 288 K. The results indicate that the surface IR emissivity does not have a significant impact on the BWIL over the selected region (not shown). Because of the strong absorption of water vapor in the water vapor channels, the surface radiation information can hardly reach the top of the atmosphere.

### 3.2. Seasonal Characteristics of Himawari-8 BWIL

**Figure 7:**
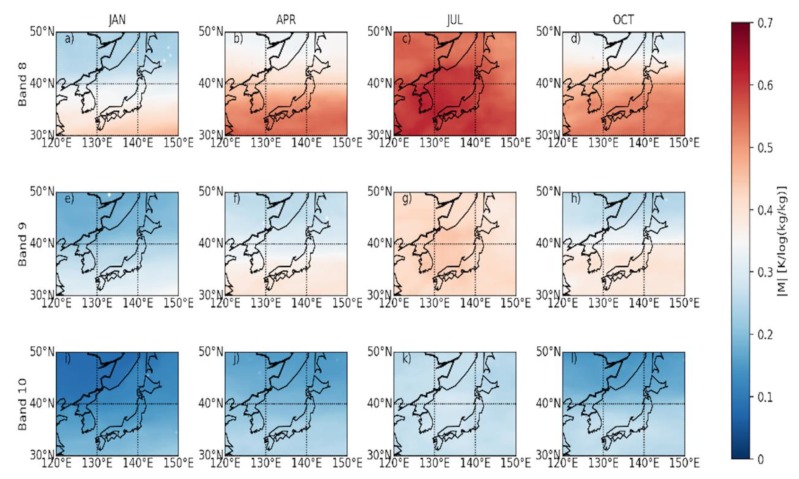
The mean |M| over the typical region of East Asia in January, April, July and October. (**a**)–(**d)** are for Band 8; (**e**)–(**h**) are for Band 9; and (**i**)–(**l**) are for Band 10. Units are K/log(kg/kg).

Analyzing the climate and area distribution characteristics of BWIL can improve the comprehension and application of the water vapor imagery from Himawari-8. Figure 7a–l show the monthly mean results of |M| at each grid over the typical region of East Asia based on the ERA-interim dataset at 00, 06, 12 and 18 UTC in January, April, July and October. From the perspective of time-evolution, |M| increases from January to July and decreases from July to October, reaching a maximum in July for all three WV bands. For a fixed month, the value of |M| always decreases from Band 8 to Band 10. The value of |M| is about 0.2 ~ 0.6 for Band 8, while it is about 0.17 ~ 0.45 for Band 9 and just about 0.07 ~ 0.3 for Band 10, respectively. Furthermore, |M| south of the chosen region is generally larger than that in the north, which may be explained by different water vapor amounts in the south and the north. 

**Figure 8:**
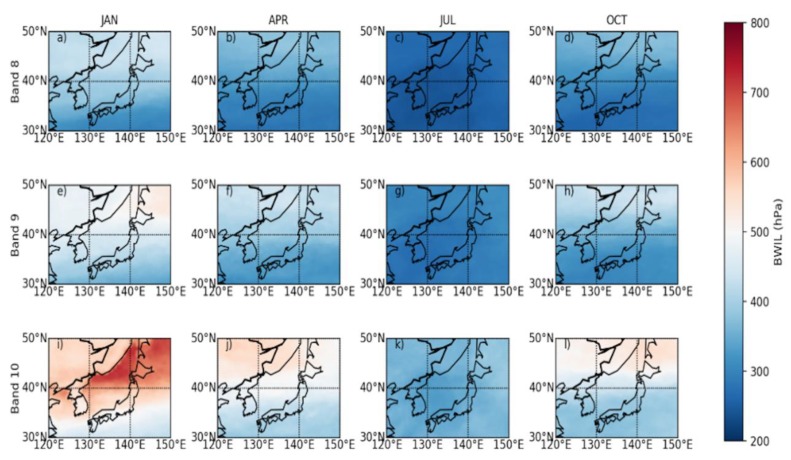
The mean height of BWIL over the typical region of East Asia in January, April, July and October. (**a**)–(**d**) are for Band 8; (**e**)–(**h**) are for Band 9; and (**i**)–(**l**) are for Band 10. Units are hPa.

Figure 8 a–l depict the monthly mean height of the BWIL. It indicates that from the perspective of time-evolution, the height of BWIL rises from January to July and descends from July to October, reaching the highest in July for all three bands. The height of BWIL for Band 8 is about 237.38 ~ 447.81 hPa, while it is about 264.53~535.95 hPa for Band 9 and about 328.66~724 hPa for Band 10. This may be connected to the seasonal distribution of moisture influenced by monsoon. Generally, winter monsoon prevails in East Asia in January, so the air over the selected region will be dry and cold [38]; instead, summer monsoon tends to transport warm and humid air flow from the ocean to the target area [39], leading to a higher BWIL. From the vertical perspective, the height of BWIL always decreases from Band 8 to Band 10, which is coincident with the characteristics of BWIL based on the U.S. standard atmosphere profile. Additionally, the inhomogeneous horizontal distributions of the height of BWIL are shown, which may stem from the lower moisture in the north and higher WV in the south transported by monsoon, as well as from the different land-sea distributions in the northern area and the southern area (there are more sea areas in the south and fewer in the north).

### 3.3. Land-Sea Variation of BWIL

**Figure 9:**
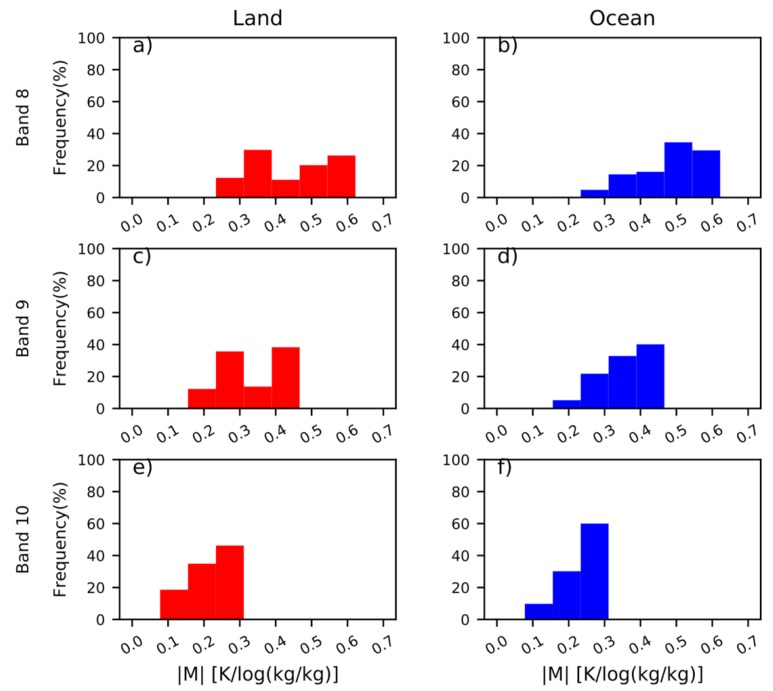
The frequency of distribution of |M| over land and ocean for (**a, b**) Band 8, (**c, d**) Band 9 and (**e, f**) Band 10, respectively.

As a result of sparse observation means at sea, it is important to apply geostationary satellites better in order to capture water vapor information from the sea [40]. To achieve this, we explore the differences in |M| and the height of BWIL between land and sea based on the ERA-interim atmospheric profiles. Figure 9a-f display the frequency distribution of |M| from the three WV bands over ocean and land. For the same band, |M|’s range over the land is similar to that of the sea, yet the range over the sea is slightly wider than that over land. 

**Figure 10:**
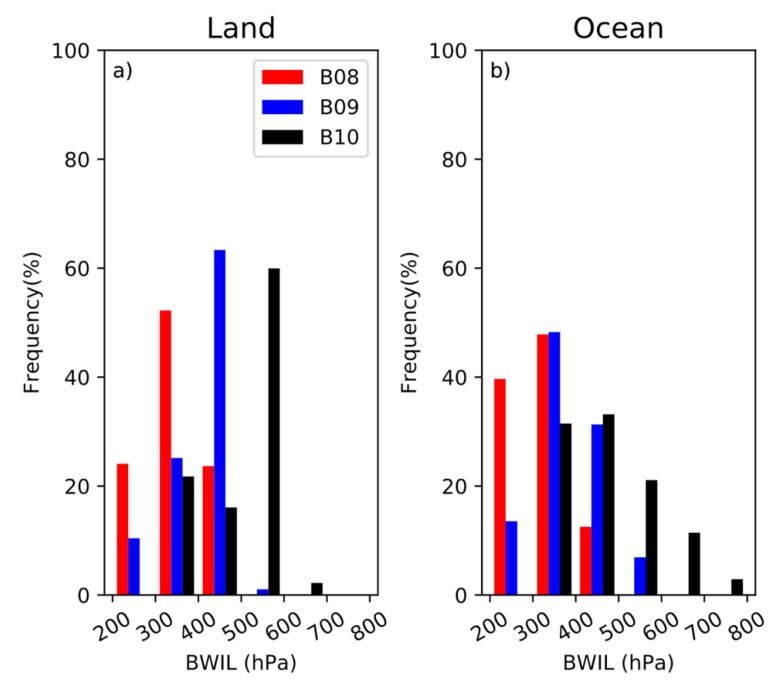
(**a**) The frequency of distribution of the height (hPa) of BWIL over the land for Band 8, Band 9 and Band 10. (**b**) The frequency of distribution of the height (hPa) of BWIL over the sea for Band 8, Band 9 and Band 10.

Figure 10 reveals the frequency distribution of BWIL over the ocean and the land. For both land and ocean, the BWIL’s height for Band 8 is the highest while it is lowest for Band 10, which is consistent with the results in Figure 3. Specifically, the proportion of small values decreases from Band 8 to Band 10 (the higher the proportion of the small values is, the higher the BWIL is). The distribution range of the sea is almost the same as that of the land in Band 8 and Band 9, while it is slightly wider than the land in Band 10. The annual mean height of the BWIL differs a lot between the land and the sea. On the land area, the mean heights are 339.90 hPa, 391.40 hPa and 481.03 hPa for Band 8, Band 9 and Band 10, respectively. Meanwhile, the mean values over the sea are 311.53 hPa, 359.01 hPa and 450.45 hPa for Band 8, Band 9 and Band 10, respectively. Therefore, the mean heights of the BWIL over the sea of the three WV Bands are all higher than those over the land. 

**Figure 11:**
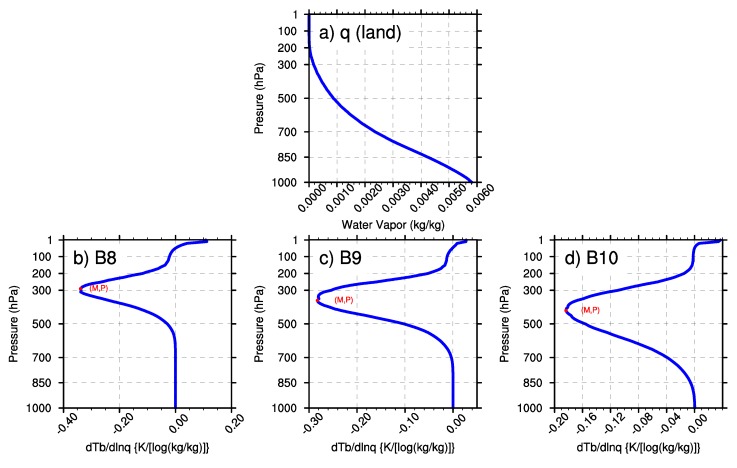
(**a**) The mean water vapor profile over the land area in the typical region of East Asia. (**b**)–(**d**) Corresponding Jacobian functions (M) and BWIL (P) of Band 8, Band 9 and Band 10, respectively.

**Figure 12:**
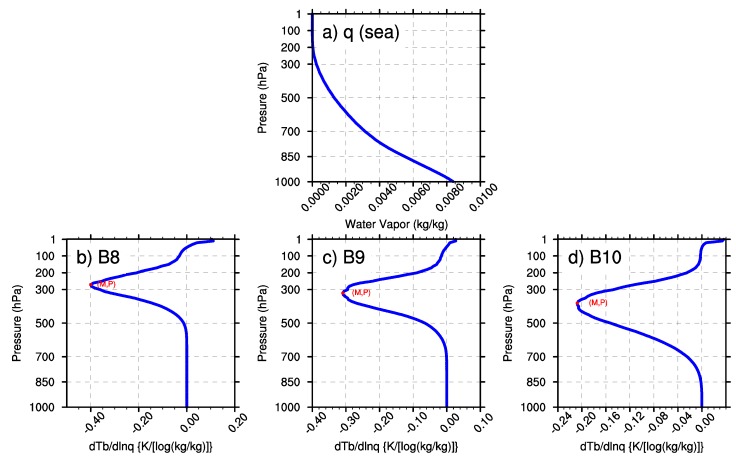
(**a**) The mean water vapor profile over the sea area in the typical region of East Asia. (**b**)–(**d**) Corresponding Jacobian functions (M) and BWIL (P) of Band 8, Band 9 and Band 10, respectively.

To make a physical and meteorological interpretation, all profiles of the ocean and the land in January, April, July and October are extracted and averaged, and the results are shown in Figure 11 and Figure 12. The extracted average water vapor profiles of the land and the sea show that the annual average water vapor over the sea is much more abundant than that over the land, so the absorption cross-section and the optical thickness are also larger, which will cause a stronger absorption effect. Consequently, the BWIL over the sea area is higher than that over the land area.

To illustrate the general characteristics of |M| and the height of BWIL (represented by P) over the ocean and the land, their mean values in January, April, July and October are shown in Table 1 and Table 2. The tables show that the mean value of |M| over the ocean is always greater than that over the land, and that the mean value P is often higher than that over the land (except for January and July for Band 10, where the BWIL heights over the sea and the land are nearly the same). Furthermore, the seasonal variation of |M| and P due to monsoon (in Section 4) also emerges. 

### 3.4. The Assessment of ERA-interim

Since the characteristics and the values of |M| and P over the selected region have been acquired, we can assess the ERA-interim dataset to figure out the applicability of its water vapor at different altitudes. In order to compare the brightness temperatures from the satellite observation and model simulation under a clear sky condition, a type of reliable cloud product with a temporal resolution of 6 h and a spatial resolution of 2 km × 2 km is used to discriminate a clear sky [35]. In 2016, the average monthly water vapor content in the chosen region reached the maximum in July, so we assess the water content of the ERA-interim dataset in July 2016 as an example. Water vapor below 400 hPa of the ERA-interim dataset has been compared with that observed by COSMIC GPS RO [41], but the assessment by Himawari-8 WV bands are more advantageous in higher pressure layers.

**Figure 13:**
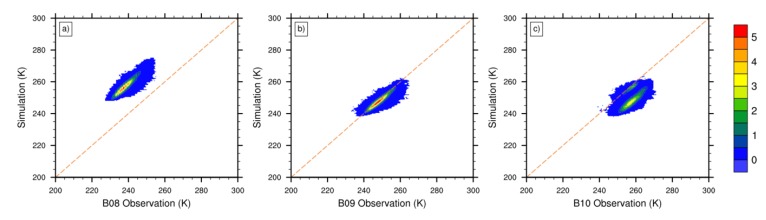
The joint probability distribution (PDF) of the simulated and observed brightness temperatures of **(a)** Band 8, **(b)** Band 9 and **(c)** Band 10 in July 2016; more than seven million pixels are included. Units are ‰.

The analysis of BWIL suggests that, in July 2016, the BWIL range is 280 ~ 240 hPa for Band 8, 320~260 hPa Band 9 and 394~328 hPa for Band 10. Figure 13 compares the simulated and observed brightness temperatures of all clear-sky pixels (more than seven million) for the three WV bands over the selected region in July 2016 though the joint probability density distribution (PDF). The assessment of Band 8 in Figure 13a shows that the brightness temperatures simulated by ERA-interim are significantly higher than those observed by satellite. This indicates that ERA-interim obviously underestimates water vapor from approximately 280 hPa to 240 hPa. The result of Band 9 in Figure 13b is generally satisfactory because the satellite observations and ERA-interim simulations are approximately equal to each other. This proves that the water vapor of ERA-interim on the corresponding BWIL pressure layers (320~260 hPa) is relatively accurate. The result of Band 10 in Figure 13c reveals that most simulated brightness temperatures are smaller than the satellite-observed BTs, which is caused by ERA-interim’s overestimation of the water vapor content on the lower pressure layers, namely 394~328 hPa. 

**Figure 14:**
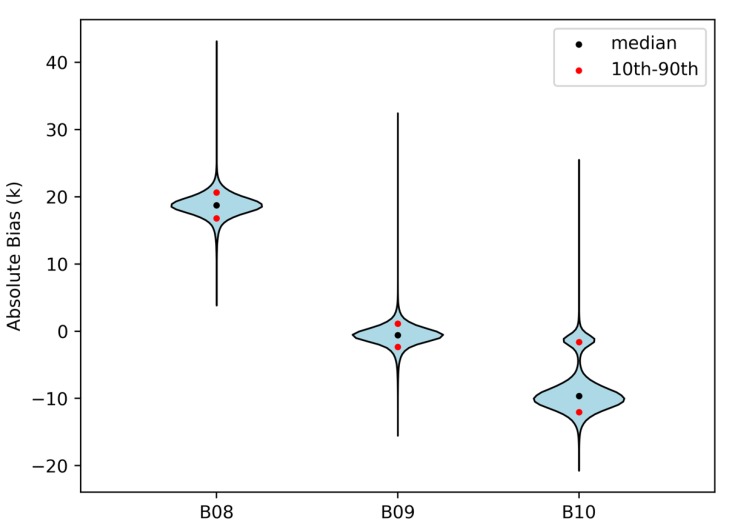
The violin plot of absolute biases between the AHI observations and simulated brightness temperatures for Band 8, Band 9 and Band 10.

Furthermore, the corresponding statistical error parameters (i.e., the mean, maximum, minimum and root mean squared error of the brightness temperature differences between the AHI observations and simulated brightness temperatures) are listed in Table 3. The maximum mean error (17.80 K for Band 8) and the maximum mean error (–0.63 K for Band 9) also indicate the same consequences obtained from Figure 13. The results of the root mean square error (RMSE) in Table 3 are another evidence that, for July 2016, the simulated brightness temperatures of Band 9 are the closest to the observed ones, followed by Band 10 and Band 8. Consequently, it can be learned that ERA-interim water vapor performs poorly above 240 hPa and below 320 hPa, while it performs comparatively better at 320~260 hPa. As a supplementary explanation, Figure 14 shows the violin plot of absolute biases between the AHI observations and simulated brightness temperatures for each WV absorption channel, indicating the complete distribution of the absolute biases. Generally, the data distributions of the three bands are centralized. The medians of the three bands are 18.7 K, –0.6 K and –9.6 K, respectively. Moreover, the absolute bias values between 16.8 K and 20.6 K account for 80% for Band 8, and the range for Band 9 is –2.4~1.1 K. The values between –12~–1.6 K account for 80% for Band 10.

## 4. Conclusions and Future Work

This work studies the BWIL of the three WV bands of Himawari-8 over a typical region of East Asia, applying the ERA-interim reanalysis dataset from 2016 and the U.S. standard atmospheric profile to calculate the Jacobian functions of water vapor. The surface emissivity is taken from MODIS. The numerical value |M| of the minimum of Jacobian function and P (the height of BWIL) are taken into consideration to explore the characteristics of BWIL. The multiple WV bands on Himawari-8 can detect moisture information in multiple atmospheric pressure layers. The main conclusions are as follows:

(1) The height of BWIL descends from Band 8 to Band 10 because of the different absorption effects of different WV bands. Furthermore, P and |M| are influenced by the water vapor profile and satellite zenith angle, yet the surface IR emissivity hardly affects the two parameters.
(2) The height of BWIL rises from winter to summer and falls from summer to autumn due to moisture transport by monsoon. Furthermore, with more water vapor, the BWIL over the sea area is higher than that over the land area.
(3) The ERA-interim water vapor profile has different performances in different pressure layers. In this study, for July 2016, ERA-interim performs well at 320 ~ 260 hPa according to the assessment of Band 9. However, ERA-interim tends to underestimate the water vapor content at 280~240 hPa and overestimate moisture at 394~328 hPa.

In this research, we merely assess the moisture performance of the ERA-interim reanalysis dataset. However, it is helpful to analyze BWILs and the uncertainties of moisture of different reanalysis datasets with multiple WV bands that can sense water vapor information at different pressure layers on Himawari-8. 

## Figures and Tables

**Table 1:** The mean |M| over land and ocean in January, April, July and October of Band 8, Band 9 and Band 10. Units are K/log(kg/kg).

	JAN		APR		JUL		OCT	
	Land	Ocean	Land	Ocean	Land	Ocean	Land	Ocean
Band 8	0.27	0.34	0.39	0.47	0.56	0.58	0.39	0.47
Band 9	0.20	0.26	0.29	0.34	0.42	0.42	0.30	0.36
Band 10	0.10	0.16	0.19	0.21	0.26	0.27	0.18	0.23

**Table 2:** The mean P over land and ocean in January, April, July and October of Band 8, Band 9 and Band 10. Units are hPa.

	JAN		APR		JUL		OCT	
	Land	Ocean	Land	Ocean	Land	Ocean	Land	Ocean
Band 8	413.41	372.34	351.22	317.82	258.79	251.97	336.17	303.99
Band 9	469.26	433.21	409.05	366.13	295.05	289.44	397.27	347.27
Band 10	560.57	563.01	520.99	451.42	359.26	359.42	483.31	427.96

**Table 3:** Mean value, maximum value, minimum value and root mean square error of the absolute errors (simulations–observations) of three WV bands in July 2016. Units are K.

	Band 8	Band 9	Band 10
Mean	18.70	–0.63	–8.42
Max	43.14	32.43	25.50
Min	3.82	–15.54	–20.75
RMSE	18.78	1.66	9.36

## References

[1] Held I.M., Soden B.J. (2000). Water Vapor Feedback and Global Warming. Annu. Rev. Energy Environ..

[2] Pierrehumbert T.R. (2002). The hydrologic cycle in deep-time climate problems. Nature.

[3] Trenberth K.E., Fasullo J.T., Kiehl J. (2009). Earth’s Global Energy Budget. Bull. Am. Meteorol. Soc..

[4] Soden B.J., Held I.M. (2006). An Assessment of Climate Feedbacks in Coupled Ocean–Atmosphere Models. J. Clim..

[5] Schmetz J., Klaes D., König M., Holmlund K. (2007). Monitoring weather and climate with the Meteosat and Metop satellites. Revista de Teledetección.

[6] Morel P., Desbois M., Szejwach G. (1978). A new insight into the troposphere with water vapor channel of MeteoSatt. Bull. Am. Meteorol. Soc..

[7] Zhang W., Xu J., Dong C., Yang J. (2006). China’s Current and Future Meteorological Satellite Systems. Earth Sci. Satell. Remote Sens..

[8] Kurino T. (2012). Future Plan and Recent Activities for the Japanese Follow-on Geostationary Meteorological Satellite Himawari-8/9. Proceedings of the 2012 AGU Fall Meeting.

[9] Iwabuchi H., Putri N.S., Saito M., Tokoro Y., Sekiguchi M., Yang P., Baum B.A. (2018). Cloud Property Retrieval from Multiband Infrared Measurements by Himawari-8. J. Meteor. Soc. Jpn.

[10] Bessho K., Date K., Hayashi M., Ikeda A., Imai T., Inoue H., Kumagai Y., Miyakawa T., Murata H., Ohno T. (2016). An Introduction to Himawari-8/9—Japan’s New-Generation Geostationary Meteorological Satellites. J. Meteorol. Soc. Jpn..

[11] Min M., Li J., Wang F., Liu Z., Menze W.P. (2020). Retrieval of cloud top properties from advanced geostationary satellite imager measurements based on machine learning algorithms. Remote Sens. Environ..

[12] Min M., Bai C., Guo J., Sun F., Liu C., Wang F., Xu H., Tang S., Li B., Di D. (2019). Estimating Summertime Precipitation from Himawari-8 and Global Forecast System Based on Machine Learning. IEEE Trans. Geosci. Remote Sens..

[13] Zhang T., Zang L., Wan Y., Wang W., Zhang Y. (2019). Ground-level PM2.5 estimation over urban agglomerations in China with high spatiotemporal resolution based on Himawari-8. Sci. Total. Environ..

[14] Liu Z., Min M., Li J., Sun F., Di D., Ai Y., Li Z., Qin D., Li G., Lin Y. (2019). Local Severe Storm Tracking and Warning in Pre-Convection Stage from the New Generation Geostationary Weather Satellite Measurements. Remote Sens..

[15] Kai-lin L., Chun-gui Z., Hong W., Xiao-chen C., Li C. (2019). Spatial and temporal distribution and variation of aerosol optical depth in coastal southeast China based on Himawari-8 satellite. J. Appl. Oceanogr..

[16] Bai W., Wu C., Li J., Wang W. (2014). Impact of Terrain Altitude and Cloud Height on Ozone Remote Sensing from Satellite. J. Atmos. Oceanic Technol..

[17] Li J. (1994). Temperature and water vapor weighting functions from radiative transfer equation with surface emissivity and solar reflectivity. Adv. Atmos. Sci..

[18] Li J., Wolf W.W., Menzel W.P., Zhang W., Huang H.-L., Achtor T.H. (2000). Global Soundings of the Atmosphere from ATOVS Measurements: The Algorithm and Validation. J. Appl. Meteorol..

[19] Chen Y., Han Y., Delst P.V., Weng F. (2010). On water vapor Jacobian in fast radiative transfer model. J. Geophys. Res. Atmos..

[20] Zeng Q. (1974). Principle of Atmospheric Infrared Remote Sensing.

[21] Soden B.J., Bretherton F.P. (1993). Upper tropospheric relative humidity from the GOES 6.7 µm channel: Method and climatology for July 1987. J. Geophys. Res. Atmos..

[22] Soden B.J., Bretherton F.P. (1994). Evaluation of water vapor distribution in general circulation models using satellite observations. J. Geophys. Res. Atmos..

[23] Soden B.J., Bretherton F.P. (1996). Interpretation of TOVS water vapor radiances in terms of layer-average relative humidities: Method and climatology for the upper, middle, and lower troposphere. J. Geophys. Res. Atmos..

[24] Soden J.B. (2000). The diurnal cycle of convection, clouds, and water vapor in the tropical upper troposphere. Geophys. Res. Lett..

[25] Huang Y., Wang M., Mao J. (2004). Retrieval of upper tropospheric relative humidity by the GMS-5 water vapor channel: A study of the technique. Adv. Atmos. Sci..

[26] Petersen A.R., Uccellini L.W., Chesters D., Mostek A., Keyser D. (1983). The use of VAS satellite data in weather analysis, prediction, and diagnosis. Nat. Weather Dig..

[27] Poc M.M., Roulleau M., Scott N.A., Chedin A. (1980). Quantitative Studies of Meteosat Water-Vapor Channel Data. J. Appl. Meteorol..

[28] Di D., Li J., Ai Y., Lu N., Shi W. (2016). Geostationary satellite-based 6.7 μm band best water vapor information layer analysis over the Tibetan Plateau. J. Geophys. Res. Atmos..

[29] Zhang F., Zhu M., Li J., Li W., Di D., Shi Y.-N., Wu K. (2019). Alternate Mapping Correlated k-Distribution Method for Infrared Radiative Transfer Forward Simulation. Remote Sens..

[30] Dee D.P.U.S., Simmons A., Berrisford P., Poli P., Kobayashi S., Andrae U., Balmaseda M., Balsamo G., Bauer D.P. (2011). The ERA-Interim reanalysis: Configuration and performance of the data assimilation system. Q. J. Roy. Meteor. Soc..

[31] Wan Z.L.Z.-L. (1997). A physics-based algorithm for retrieving land-surface emissivity and temperature from EOS/MODIS data. IEEE Trans. Geosci. Remote Sens..

[32] Wan Z.H.S., Hulley G. MYD11C2 MODIS/Aqua Land Surface Temperature/Emissivity 8-Day L3 Global 0.05 Deg CMG V006. NASA EOSDIS LP DAAC: 2015. https://lpdaac.usgs.gov/products/myd11c2v006/.

[33] Wan Z. (2008). New refinements and validation of the MODIS land-surface temperature/emissivity products. Remote Sens. Environ..

[34] McClatchey R.A., Fenn R.W., Selby J.E.A., Volz F.E., Garing J.S. (1972). Optical Properties of the Atmosphere. Air Force Rep..

[35] Xi W., Min M., Wang F., Guo J., Li B., Tang S. (2019). Intercomparisons of Cloud Mask Products Among Fengyun-4A, Himawari-8, and MODIS. IEEE Trans. Geosci. Remote Sens..

[36] Fischer H., Eigenwillig N., Müller H. (1989). Information Content of METEOSAT and Nimbus/THIR Water Vapor Channel Data: Altitude Association of Observed Phenomena. J. Appl. Meteorol..

[37] Li J., Zhou F., Zeng Q. (1994). Simultaneous Non-linear Retrieval of Atmospheric Temperature and Absorbing Constituent Profiles from Satellite Infrared under Radiances. Adv. Atmos. Sci..

[38] Zhou W., Chan J.C.L., Chen W., Ling J. (2009). Synoptic-scale controls of persistent low temperature and icy weather over southern China in January 2008. Mon. Wea. Rev..

[39] Yihui D., Chan J.C.L. (2005). The East Asian summer monsoon: An overview. Meteorol. Atmos. Phys..

[40] Agarwal S., Al Biltar A., An L., Arvidson T., Liang S. (2018). Comprehensive Remote Sensing.

[41] Kishore P., Ratnam M.V., Namboothiri S.P., Velicogna I., Basha G., Jiang J.H., Igarashi K., Rao S.V.B., Sivakumar V. (2011). Global (50°S–50°N) distribution of water vapor observed by COSMIC GPS RO: Comparison with GPS radiosonde, NCEP, ERA-Interim, and JRA-25 reanalysis data sets. J. Atmos. Solar Terr. Phys..

